# Intermittent fasting and bone health: a bone of contention?

**DOI:** 10.1017/S0007114523000545

**Published:** 2023-11-14

**Authors:** David J. Clayton, Ian Varley, Maria Papageorgiou

**Affiliations:** 1 Musculoskeletal Research Group, Nottingham Trent University, Clifton Campus, Nottingham, UK; 2 Division of Bone Diseases, Geneva University Hospitals and Faculty of Medicine, University of Geneva, Geneva, Switzerland

**Keywords:** Intermittent fasting, Alternate-day fasting, Time-restricted eating, Weight loss, Bone health, Bone turnover markers, Bone mass

## Abstract

Intermittent fasting (IF) is a promising strategy for weight loss and improving metabolic health, but its effects on bone health are less clear. This review aims to summarise and critically evaluate the preclinical and clinical evidence on IF regimens (*the 5:2 diet*, alternate-day fasting (ADF) and time-restricted eating (TRE)/time-restricted feeding and bone health outcomes. Animal studies have utilised IF alongside other dietary practices known to elicit detrimental effects on bone health and/or in models mimicking specific conditions; thus, findings from these studies are difficult to apply to humans. While limited in scope, observational studies suggest a link between some IF practices (e.g. breakfast omission) and compromised bone health, although lack of control for confounding factors makes these data difficult to interpret. Interventional studies suggest that TRE regimens practised up to 6 months do not adversely affect bone outcomes and may even slightly protect against bone loss during modest weight loss (< 5 % of baseline body weight). Most studies on ADF have shown no adverse effects on bone outcomes, while no studies on the ‘5–2’ diet have reported bone outcomes. Available interventional studies are limited by their short duration, small and diverse population samples, assessment of total body bone mass exclusively (by dual-energy X-ray absorptiometry) and inadequate control of factors that may affect bone outcomes, making the interpretation of existing data challenging. Further research is required to better characterise bone responses to various IF approaches using well-controlled protocols of sufficient duration, adequately powered to assess changes in bone outcomes and designed to include clinically relevant bone assessments.

## Graphical abstract

**Figure:**
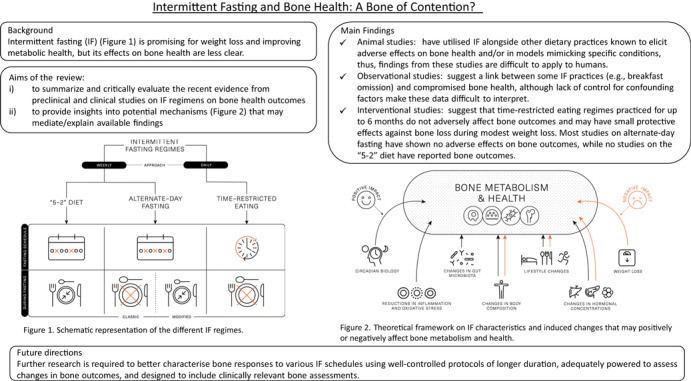

Fasting has been practised for centuries during religious events (e.g. Ramadan), and historical data also exist on fasting during hunger strikes, famine and therapeutic fasts for the treatment of morbid obesity^(1)^. However, short-term fasting has been modernised in the past 10–15 years as an unconventional approach to weight loss and improving metabolic health, with the terms *‘intermittent fasting’* and the *‘5:2 diet’* being popularised in the UK following the release of Michael Mosely’s 2013 book (*‘the Fast Diet’*). Today, intermittent fasting (IF) is a common method of reducing energy consumption, with 1 in 4 of American adults surveyed in a recent poll reporting that they consider using or have tried IF^(2)^ and publications related to IF increasing exponentially every year over the past decade (2011:19 publications, 2021:375 publications; source: Pubmed^R^, accessed 18th August 2022). There is evidence that IF can lead to weight loss and can elicit positive health-related outcomes, such as improved insulin sensitivity, blood lipid profile and lower blood pressure^(3–6)^. What sets IF apart from traditional diets involving daily energy restriction is that IF involves either complete or substantial energy restriction within defined temporal windows and permits adequate or *ad libitum* eating outside of these windows. Despite IF being shown to have a variety of health benefits, there are limited data related to the impact of IF on other bodily systems including the skeletal system.

Weight loss achieved by continuous energy restriction alone (i.e. mild to severe energy restrictions with or without micronutrient supplementation) and/or in combination with exercise has been shown to reduce bone mass and negatively affect bone microstructure^(7–12)^. Several mechanisms have been proposed to explain these effects including mechanical unloading, nutrient deficiencies and endocrine changes^(13–15)^. It remains uncertain whether IF is a dietary intervention with similarly undesirable bone effects or if specific characteristics of the IF schedules may have positive effects on bone and counteract/prevent the bone changes seen with conventional weight loss approaches. For example, IF is suggested to affect metabolism by repeatedly alternating fixed periods of prolonged fasting (i.e. catabolism) with shortened periods of eating (i.e. anabolism) and/or by synchronising eating behaviours to endogenous circadian rhythms^(3,16,17)^. Better metabolic control^(18,19)^ and circadian alignments^(20)^ are considered beneficial for skeletal health. Interestingly, the net effects of the different characteristics and effects of IF interventions on bone heath are poorly understood.

The purpose of this review is to (i) summarise and critically evaluate the recent evidence from preclinical and clinical (epidemiological and interventional) studies on IF regimens on bone health outcomes in adults, (ii) provide insights into potential mechanisms that may mediate/explain available findings and (iii) identify limitations and knowledge gaps of current research, with the goal to provide directions for future research. Hence, it is envisaged that this review will guide the design of future research in this area and will help practitioners/individuals who aim to follow these regimens reach informed decisions.

## Definition of IF regimens

**Fig. 1.**
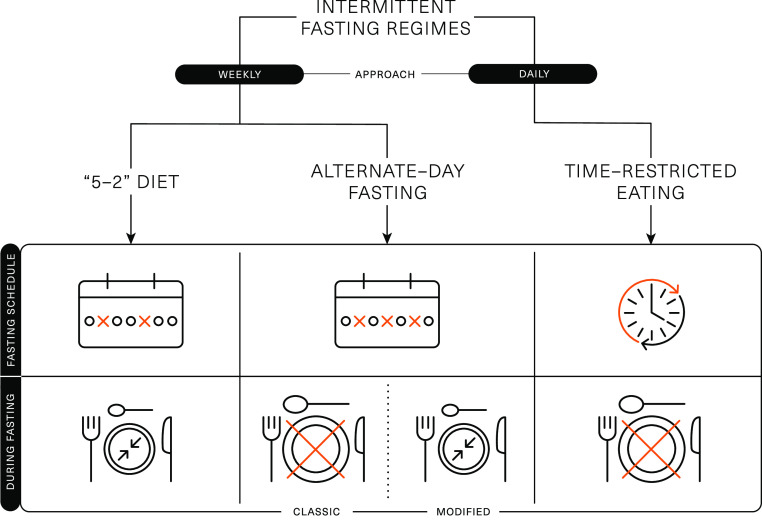
Schematic representation of the different intermittent fasting regimens.

Several different methods are encompassed under the broad term of IF, which represents a challenge with interpreting data from the literature. *The 5:2 diet*, alternate-day fasting (ADF), alternate-day modified fasting and time-restricted eating (TRE)/time-restricted feeding are the most adopted and researched types of IF regimens (Fig. 1).

*The ‘5:2’ diet* is framed around a 7-d rolling period (typically a week), wherein severe energy restriction (consuming ∼25 % of energy requirements) is imposed on 2 d of the week and *ad libitum* eating is permitted on the remaining 5 d. This method of IF has been shown to acutely reduce energy intake^(21,22)^ and lead to 4–7 % weight loss over 8–52 weeks^(3,4,23–26)^. Weight loss and body compositional changes resulting from *‘5:2’* dieting are broadly comparable to continuous daily energy restrictions and elicit similar improvements in metabolic health^(4,23,27)^. There is some evidence that the effects of *‘5:2’* dieting are more pronounced if the 2 d of severe energy restriction are undertaken consecutively. This approach was shown to achieve greater improvements in insulin sensitivity and post-prandial lipaemia, compared with daily energy restriction resulting in similar weight loss and fat mass reductions^(23,28)^. Some of the metabolic adaptations related to IF are thought to be due to prolonged periods of complete fasting; however, periods of fasting are curtailed on *the ‘5:2’ diet* due to the permission of a small meal on ‘fasting’ days, so the duration of uninterrupted fasting is usually unknown.

ADF involves a day of fasting alternated with a day of adequate or *ad libitum* eating. While ADF has been shown to achieve weight loss^(29,30)^, day-long periods of complete fasting on alternate days have been shown to reduce lean mass to a greater extent than traditional energy restriction^(30)^ and have been associated with lack of adherence^(31)^. Due to these potential negative effects on body composition and compliance challenges, alternate-day modified fasting was devised as a hybrid of ADF and *the 5:2 diet*, which permits some energetic intake (usually 25 % of energy requirements; ∼2092 kJ or 500 kcal) on restricted (or ‘fast’) days. Alternate-day modified fasting has been shown to achieve 4–8 % weight loss over 8–52 weeks^(3,6,29,32–34)^, which is comparable to the weight loss achieved by traditional energy restriction applied daily. There is also some evidence that alternate-day modified fasting can improve markers of metabolic health, including insulin sensitivity and blood lipids^(6,35)^.

TRE is a daily approach to IF and is based around prescribed fasting and eating windows within each day. This approach restricts food intake to short daily windows (4–12 h), thereby extending the overnight fast to at least 12 h^(3,16)^. TRE can be achieved by skipping a meal (breakfast or dinner) or shifting the time of meals (e.g. delaying breakfast and/or advancing dinner)^(36,37)^. Evidence for breakfast omission is mixed, with some studies associating regular breakfast omission with a higher BMI and an increased risk of chronic diseases^(38,39)^. However, short-term empirical studies indicate breakfast omission may be an effective way to reduce daily food intake^(40–42)^. There is some evidence that evening fasting can improve a range of health outcomes^(43)^, even in the absence of weight loss^(44)^. This may be related to several metabolic markers displaying circadian variation that ameliorate in the morning and decline towards the evening. As such, when the fasting period is implemented may profoundly influence the effects of TRE.

## Assessment of bone health and fragility

Bone mass or density, turnover, structure and strength can be evaluated (or estimated) using direct bone measurement or surrogate endpoints or biomarkers. For a detailed description of the available techniques, the reader is directed in previous reviews^(45–47)^. In brief, dual-energy X-ray absorptiometry (DXA) is the most frequently used technique to determine bone mineral content (BMC) and areal bone mineral density. To date, DXA remains the gold standard for diagnosing and monitoring osteoporosis as it has been shown to be linearly associated with fracture risk^(48,49)^. Peripheral quantitative computed tomography is a three-dimensional technique that can be used to assess volumetric bone mineral density and bone geometry at appendicular skeletal sites^(46)^. It allows the distinction between trabecular and cortical bone and provides measures of total and cortical bone area, cortical thickness and estimates of bone strength. These instruments are mostly used for research purposes and can be further combined with other techniques to estimate bone mechanical characteristics. The assessment of fracture risk is the ultimate outcome in bone research. Nevertheless, incident fractures are commonly evaluated in observational studies but rarely in clinical trials^(46,47)^. This is because of the large sample sizes required and the long periods of follow-up needed to capture their development/manifestation.

Alternatively, bone turnover markers (BTMs) are surrogate markers that allow the determination of changes in bone formation and bone resorption rates and may help to monitor the effects of shorter-term interventions^(45,46)^. BTMs reflect acute changes in bone metabolic activity and require a shorter period of assessment than a serial collection of BMD/BMC. BTMs are classified as indices of bone resorption or formation. The most widely used bone resorption markers are products of type I collagen breakdown generated during bone resorption (C-terminal cross-linking telopeptide of type I collagen (CTX), NTX, pyridinium cross-links) or indicators of osteoclast activity (tartrate-resistant acid phosphatase). Bone formation markers include products of post-translational processing of type I collagen molecules (procollagen type I N propeptide (P1NP), procollagen type I C propeptide (P1CP)), matrix proteins (osteocalcin) or enzymes (bone-specific alkaline phosphatase) released in the circulation from osteoblasts during their activity of bone matrix synthesis. This is particularly useful given the majority of the studies that have assessed the effects of IF on bone health are currently of short duration (< 6 months).

## Search strategy

A literature search was conducted using MEDLINE database until the 30th of September 2022. We included animal studies, human observational (cross-sectional and longitudinal) and interventional studies in adult populations. Relevant studies were selected using a combination of keywords for skeletal health outcomes (bone, bone mineral density, osteoporosis, fracture, bone turnover or bone remodelling) and IF, alternate-day, *‘5:2’* diet, time-restricting eating or feeding as explanatory variables. Additional studies were identified by a manual search of bibliographic references in original papers and reviews.

## Studies affecting the effects of intermittent fasting on bone health

### Animal studies

The animal studies that have investigated the effects of IF on bone health tend to utilise IF in addition to other practices (e.g. high-fat or ketogenic diet)^(50,51)^ that are known to elicit negative effects on bone health^(52,53)^ and/or in models of specific conditions (e.g. Alzheimer’s disease-induced oestrogen deficient rats^(51)^, glucocorticoid-induced osteoporosis animal model^(54)^).

One study investigated the effects of a ketogenic diet with or without ADF for 12 weeks in rats^(50)^. ADF while consuming a ketogenic diet inhibited osteoclast proliferation and osteogenic differentiation compared with a daily ketogenic diet (without ADF), but this did not translate in differences in bone structure. In the same study, compared with a control group consuming a standard diet, the ADF ketogenic diet caused a decrease in bone strength and impaired parameters of cancellous (lower trabecular total mineral density, bone volume/total volume, trabecular number and separation) and cortical bone (lower total area, bone area and cortical thickness), although the same adverse effects were also shown in rats consuming a daily ketogenic diet. Furthermore, the control group had lower levels of bone resorption markers and higher levels of bone formation markers than both the daily ketogenic diet and ADF ketogenic diet groups. While IF may contribute to the negative effects on bone characteristics, it is impossible to isolate the effect of the ketogenic diet, which appears to have largely driven the negative bone effects shown in this study.

Time-restricted feeding (3 h of feeding per day for 4 weeks) alongside a high-fat diet (dietary fat provided ∼46 % of total energy) was found to reduce femoral BMD compared with an *ad libitum* high-fat eating group, in Alzheimer’s disease-induced oestrogen deficient rats^(51)^. The reason for time-restricted feeding having a negative effect on bone may relate to the high-fat composition of the diet^(55)^. A greater amount of fat deposition, in the high-fat diet, could cause a reduction in osteoblasts as a result of bone marrow mesenchymal cells differentiation favouring adipogenesis ultimately having a negative effect on bone mass^(56)^. Ultimately, this study found that time-restricted feeding was not able to protect bone against the negative effects of a high-fat diet.

Animal studies assessing the effects of IF on bone health are useful in providing mechanistic insights. However, combining IF with other dietary practices expected to elicit negative effects on bone may have confounded these findings. In addition, variation in the type of IF adopted and the study implementation in models mimicking specific medical conditions indicate caution when interpreting the effects of IF on bone characteristics. This underpins the need for further animal work to provide mechanistic insights into IF scenarios more applicable to humans.

### Observational studies

Observational studies investigating IF and bone health are largely lacking. To our knowledge, in the only available cross-sectional analysis, no differences were seen in total body BMC or lumber spine BMD among healthy adults who were following ADF over periods ≥ 6 months and healthy controls with no history of performing ADF^(57)^. Cross-sectional studies have a number of issues when assessing bone health and IF. Their design only allows for a discrete assessment of bone health to be made, and therefore it is impossible to establish a cause–effect relationship. It is also very rare for cross-sectional studies to control/monitor confounding factors, such as exercise status and the specific IF protocols.

Currently, the long-term implication of IF on bone health is best inferred using data from studies assessing breakfast omission. In a cross-sectional analysis, young women (aged 19–25 years old) who skipped breakfast ≥ 3 times per week had lower hip BMD compared with those who consumed breakfast daily^(58)^. Furthermore, in a longitudinal study with a 3-year follow-up, young men who reported skipping breakfast (classified as breakfast consumed less often than every day) had higher odds of experiencing bone loss at the lumbar spine in comparison with men consuming breakfast daily, while no significant associations were seen for bone loss at other sites (i.e. hip) or in women^(59)^. Interestingly, breakfast omission has also been associated with various unhealthy lifestyle factors, such as smoking and increased alcohol consumption^(42)^, which also tended to be higher among those who lost bone^(59)^. Taken together, while epidemiological evidence associates breakfast omission with bone loss, it is likely that indirect factors, such as unhealthy lifestyle choices, confound the direct associations made.

### Interventional studies

Human trials exploring the effects of IF regimens on bone outcomes have only begun to emerge over the past 5 years. We identified eight randomised controlled trials (RCTs), which investigated the effects of IF on bone mass or BTMs in individuals with/without obesity and/or other metabolic disturbances (Table 1). These studies utilised ADF (*n* 3) or TRE (*n* 5) approaches, but we did not find any studies on *the ‘5–2’ diet*.

**Table 1.** Recent clinical trials (2017–to date) exploring the effects of IF regimens on outcomes of bone health

Study	Study population	Duration	Intervention description	Weight changes	Main bone findings
ADF (*n* 3)					
Barnosky *et al.*, 2017^(60)^	Overweight/obese adults aged 18–65 years, 3 groups (ADF, CER, control) ADF: *n* 21, mean ± sem age: 44 ± 2 years, BMI: 34 ± 1 kg/m^2^, 19 W/2 M CER: *n* 24, age: 44 ± 2 years, BMI: 34 ± 1 kg/m^2^, 20 W/4 M Control: *n* 17, age: 40 ± 3 years, BMI: 32 ± 1 kg/m^2^, 15W/2M	6 months	ADF: 25 % DEI fast day, alternated with 125 % DEI feast day	–7·8 ± 1·2 %	Total body BMC and BMD remained unchanged in all groups. OC, BAP and CTX did not change in any group. No differences between premenopausal or postmenopausal women for any marker
			CER: 75 % DEI every day	–8·8 ± 1·5 %	
			Control: habitual intake	NS	
Stekovic *et al.*, 2019^(57)^	Non-obese adults aged 35–65 years, 2 groups (ADF, control) ADF: *n* 29, median age (IQR): 48 (43–55) years, mean BMI (±sd): 25·5 ± 1·8 kg/m^2^, 17 W/12 M Control: *n* 28, age 51 (45–57) years, BMI: 25·4 ± 2·2 kg/m^2^, 17 W/11 M	4 weeks	ADF: *ad libitum* eating every second-day, no kcal on the fast days	–3·5 ± 1·5 kg	BMD at the lumbar spine region (derived from total body scans) ↓ after ADF but not after control. Comparing Comparison of ΔBMDs of both groups did not yield significant differences. No within or between group differences were seen for total body BMC
			Control: habitual diet	NS:–0·2 ± 1·1 kg	
Templeman *et al.*, 2021^(30)^	Lean healthy adults aged 18–65 years, 3 groups (CER 75:75, ADF 0:150, ADF 0:200) CER 75:75: *n* 12, mean ± sd age: 45 ± 6 years, BMI: 24·0 ± 1·9 kg/m^2^, 7W/5M ADF 0:150: *n* 12, age: 42 ± 11 years, BMI: 23·9 ± 2·4 kg/m^2^, 5W/7M ADF 0:200: *n* 12, age: 41 ± 14 years, BMI: 23·6 ± 2·1 kg/m^2^, 9 W/3 M	3 weeks	CER: 75 % DEI (75:75)	–1·9 ± 1·0 kg	No differences in plasma CTX pre- and post-intervention. No group differences in BMC or BMD
			ADF with ER: 24-h fasting with 150 % DEI on alternate days (0:150)	–1·6 ± 1·1 kg	
			ADF without ER: 24-h fasting 200 % DEI on alternate days (0:200)	NS: −0·5 ± 1·1 kg	
TRE (*n* 5)					
Martens *et al.*, 2019^(63^	Apparently healthy, non-obese adults, 2 crossed-over interventions (TRE, control) *n* 22, mean ± sem age: 67 ± 1 years, BMI: 24·7 ± 0·6 kg/m^2^, 12 W/10 M	6 weeks	TRF: *ad libitum* 8-h eating window	NS	Total and regional BMD were not different between conditions
			Control: habitual diet	NS	
Lowe *et al.*, 2020^(61)^	Overweight and obese adults (in-person cohort), two groups (TRE, consistent meal timing (CMT)) TRE: *n* 25, mean ± sd age: 43 ± 12 years, BMI: 31·5 ± 4·5 kg/m^2^, 12 W/13 M CMT: *n* 25, age:44 ± 11 years, BMI: 31·3 ± 3·5 kg/m^2^, 10 W/15 M	12 weeks	TRE: *ad libitum* 8-h eating window between 12.00 and 20.00 hours	–1·7 kg	A trend towards a ↑ in BMC in the TRE group (*P* = 0·09) but not in the CMT group. BMC changes did not differ between groups
			CMT: Habitual diet consumed in 3 structured meals per day	NS: 0·6 kg	
Lobene *et al.*, 2021^(62)^	Overweight and obese adults, two groups (TRE, non-TRE) TRE: *n* 11, mean ± sem age: 47 ± 4 years, BMI: 33·8 ± 2·3 kg/m^2^, 9 W/2 M non-TRE: *n* 9, age: 44 ± 4 years, BMI:34·4 ± 2·6 kg/m^2^, 8 W/1 M	12 weeks	TRE: *ad libitum* 8-h eating window	–3·7 ± 0·5 %	P1NP ↓ in both groups (time effect) with a trend towards a greater ↓ in the non-TRE group (time × group interaction, *P* = 0·07). Total body BMC ↑ in the TRE group and ↓ in the non-TRE group (time × group interaction, *P* = 0·02)
			Non-TRE: habitual diet	NS	
Kotarsky *et al.*, 2021^(67)^	Overweight/obese adults, two groups (TRE + ex, non-TRE + ex) TRE + ex: *n* 11, age: 45 ± 3 years, BMI: 29·8 ± 0·8 kg/m^2^, 9 W/2 M non-TRE + ex: *n* 10, age: 44 ± 2 years, BMI: 29·4 ± 0·8 kg/m^2^, 9 W/1 M	8 weeks	TRE + ex : *ad libitum* 8-h eating window between 12.00 and 20.00 hours + exercise (aerobic + resistance)	3·3 %	No significant time, group or time × group interaction for total body BMC or BMD
			Non-TRE + ex: habitual diet + exercise (aerobic + resistance)	NS: 0·2 %	
Papageorgiou *et al.*, 2022^(64)^	Individuals with at least one component of the metabolic syndrome, two groups (TRE, SDA) TRE: *n* 23, median (IQR) age: 47 (32, 57) years, BMI: 27·9 (25·5, 31·4) kg/m^2^, 18 W/5 M SDA: *n* 19, age: 45 (27, 50) years, BMI: 26·7 (23·8, 30·6) kg/m^2^, 14 W/5 M	6 months	TRE: *ad libitum* 12-h eating window	–0·6 kg (median)	Total cohort: no between-group differences (TRE *v*. SDA) in CTX, P1NP or total body BMC/BMD responses. Analysis by weight loss response: among weight loss responders, CTX tended to ↓ after TRE but ↑ after SDA (between-group differences *P* = 0·041), P1NP changes did not differ between groups. Total body BMC ↓ after SDA, but remained unchanged after TRE (between-group differences in weight loss responders *P* = 0·028). Among non-responders (< 0·6 kg weight loss), there were no between-group differences in bone outcomes
			SDA: 10-min counselling for healthy eating + healthy eating brochure	NS	

↓, indicates a decrease; ↑, indicates an increase; ADF, alternate-day fasting; BAP, bone-specific alkaline phosphatase; BMC, bone mineral content; BMD, bone mineral density; CER, continuous energy expenditure; CMT, consistent meal timing; CTX, *β*-carboxyterminal telopeptide of type I collagen; Δ, delta; DEI, dietary energy intake; ex, exercise; M, men; NS, non-significant; OC, osteocalcin; P1NP, procollagen type 1 N-terminal propeptide; SDA, standard dietary advice; TRE, time-restricted eating; W, women; IQR, inter-quartile range; .

The primary outcomes of the included studies were changes in body weight (*n* 3; Barnosky *et al.* ^(60)^; Lowe *et al.* ^(61)^; Kotarsky *et al.* ^(67)^), changes in body composition (*n* 1; Templeman *et al.* ^(30)^), changes in insulin sensitivity (*n* 1; Stekovic *et al.* ^(57)^), changes in components of energy balance and post-prandial metabolism (*n* 1; Templeman *et al.* ^(30)^), change in the metabolic syndrome components (*n* 1; Papageorgiou *et al.* ^(64)^) and changes in endothelium-dependent dilation (*n* 1; Martens *et al.* ^(63)^).

#### Alternate-day fasting

A study in healthy lean males and females compared the effects of ADF with net energy restriction (25 % energy deficit) with continuous energy restriction (matched 25 % energy deficit applied daily) and ADF without energy restriction over 3 weeks^(30)^. Energy restriction, however implemented, resulted in similar weight loss (∼2 kg), which was greater than the weight loss observed after ADF without energy restriction. Interestingly, while weight loss after continuous energy restriction was largely achieved by reducing body fat mass, ADF led to lesser reductions in fat mass but also a trend towards a reduction in lean mass. No significant changes were seen in the plasma concentrations of the bone resorption marker CTX or total body BMD (assessed by DXA), which may be explained by the modest changes in body weight/body composition and the short duration of the study protocol.

These results are in accordance with a 6-month RCT which compared the effects of ADF (25 % energy deficit), continuous energy restriction (25 % energy deficit applied daily) or participants’ habitual diet (control group) in individuals with overweight or obesity^(60)^. Although participants achieved significant weight loss after ADF (–7·8 (sd 1·2) %) and continuous energy restriction (–8·8 (sd 1·5) %) compared with controls, no significant changes were reported for total body BMC or BMD (by DXA) in any of the groups. Circulating levels of surrogate markers of bone formation (osteocalcin, bone alkaline phosphatase) and bone resorption (CTX) also remained unchanged in all groups.

In another RCT, non-obese adults were randomly allocated to either an ADF group or a control group maintaining their habitual diet^(57)^. After 4 weeks, the ADF group reduced their energy intake by ∼37 %, body weight by 3·5 % and trunk fat by 15 %. Although total body BMC (by DXA) was not affected in either group, BMD at the level of the lumbar spine (extracted from regional analysis of total body DXA scans) decreased on average by 0·9 % in the ADF group compared with smaller BMD reductions (0·5 %) in the control group. Notably, this decrease was only significant when analysed within the ADF group, while between-group comparison of BMD changes was not significantly different. Postmenopausal women typically experience 1 % BMD loss per year, while BMD has been shown to significantly decrease by ∼1 % over longer-term periods of milder continuous energy restrictions^(7,10,11)^. Thus, the magnitude of BMD reductions ^(57)^ within such a short timeframe, if continued, could be concerning for longer-term bone health and lifelong fragility fracture risk.

#### Time-restricted eating

Two RCT investigated the effects of TRE (8-h eating window) *v*. control (habitual diet) for 12 weeks in adults with overweight/obesity^(61,62)^. Lowe *et al.* found that the TRE group decreased their body mass from baseline by a small amount (–1·7 kg) tended to have an increase in total body BMC, while no changes were observed for body weight or bone mass in the control group^(61)^. Lobene *et al.* reported reductions in the bone formation marker P1NP when pooling data from the TRE and control groups, with a trend towards a greater P1NP reduction in the control group, and no changes in other markers of bone metabolism (NTX or PTH)^(62)^. The BTM results from Lobene *et al.* may indicate a protective response of TRE and were, to some extent, further supported by small changes in bone mass, with total body BMC decreasing in the control group but increasing in the TRE group.

Using a similar TRE protocol (16-h fasting with 8-h *ad libitum* eating), a cross-over RCT explored the feasibility and safety of a 6-week TRE intervention (*v*. control-habitual diet) in middle-aged and older individuals^(63)^. This study found that TRE had no impact on participants’ body mass, lean mass, total body or regional BMD (by total body DXA). The absence of significant changes in these bone parameters may be explained by the modest changes in lifestyle factors (i.e. participants reduced their eating window by ∼4 h but did not change their energetic intake or dietary quality) or may reflect the short follow-up of the study which was likely insufficient to detect small BMD changes.

In line with these findings, a longer-term (6 months) TRE intervention (employing a 12-h *ad libitum* eating window) had no unfavourable effects on bone metabolism (BTMs and bone-related hormones) or bone loss (total body BMC/BMD by DXA) compared with the provision of standard dietary advice for healthy eating, in individuals with at least one component of the metabolic syndrome^(64)^. Additional sub-analysis in participants who lost weight with either TRE or standard dietary advice (based on median body weight changes: ≥ 0·6 kg weight loss) found that those who lost weight by following SDA experienced a modest loss of total body BMC, which was supported by small, albeit non-significant increases in bone resorption (CTX). By contrast, when weight loss was achieved by TRE, BMC was preserved with CTX concentrations tending to decrease. These findings suggest a possible benefit of TRE on bone health during weight loss, although it should be noted that results reflect bone responses to a milder TRE intervention allowing a longer eating window than typically employed (12 h), and high inter-participant variation in body weight responses.

Exercise, particularly resistance exercise, has previously been shown to mitigate the undesirable effects of weight loss on bone and muscle^(9,65,66)^. Therefore, Kotarsky *et al.* compared the effects of 8 weeks of TRE (eating window between 12.00 and 20.00 hours) in combination with an aerobic and resistance exercise programme (TRE+ex), compared with a habitual diet with the same exercise programme (control+ex), in adults with overweight and obesity^(67)^. Both interventions induced significant energy deficits (TRE+ex: ∼300 kcal/d, control+ex: ∼250 kcal/d), but TRE+ex reduced total body mass (3·3 % *v*. 0·2 %) and fat mass (9·0 % *v*. 3·3 %) to a greater extent than control+ex. LBM tended to increase due to exercise, with no differences between groups. These changes in body weight and body composition were not accompanied by changes in total body BMC or BMD.

Despite the increasing number of interventions reporting bone outcomes, it is challenging to draw conclusions from the current studies. Overall, studies suggest that TRE regimens practised for relatively short periods (up to 6 months) do not appear to adversely affect bone outcomes and may even slightly protect bone when weight loss occurs. Similarly, most studies on ADF have shown no adverse effects on bone outcomes, while studies on the effects of the ‘*5:2’ diet* on bone are lacking. Current understanding is limited by the following factors, indicating caution when interpreting the results of existing studies and underpinning the importance of future research.

- *Short duration*: Current trials had short durations (ranging from 3 weeks to 6 months). Trial duration affects body weight, metabolic and skeletal effects. For example, longer trial durations (> 12 weeks) may be required with some methods of IF to observe clinically significant weight loss (≥ 5 % weight loss from baseline)^(3)^. The effects of dietary interventions on bone mass also need sufficient time to present^(7)^. This is because a complete cycle of bone remodelling takes 4–6 months; thus, studies are proposed to have a duration of ≥ 6 months to allow the detection of clinically meaningful changes in bone structure (by DXA or other imaging modalities)^(7)^. The very few studies that have assessed BTM responses to IF interventions suggest no changes after ADF^(60)^ or some favourable effects of TRE^(62,64)^, with these findings indicating no major bone breakdown at least in the short/medium term.
- *Bone assessments*: It is of note that all available studies have reported total body and/or regional BMC/BMD based on total body DXA assessments but have not performed scans at clinically relevant sites, that is, the hip or the lumbar spine, or evaluations of bone microstructure and fracture risk.
- *Lack of power to detect changes/differences in bone outcomes*: None of the available studies has assessed bone parameters as *a priori* outcomes (Table 1). Conversely, since most of the available trials had small sample sizes, it is likely that these studies were not powered to detect differences in bone parameters assessed as secondary outcome measures.
- *Population at high risk for bone fragility*: Overall, existing studies have been conducted in small mixed population samples, with the effects of IF regimens on bone outcomes in groups at high risk for bone fragility (i.e. elderly, postmenopausal women) remaining largely understudied. One study found that TRE was not associated with bone loss in middle-aged and older individuals, although the TRE intervention implemented did not result in pronounced lifestyle changes and had a short duration (6 weeks) (for a detailed description, see Table 1). Notably, it has been suggested that IF may not be appropriate for some population groups including children and adolescents, pregnant or breast-feeding women, individuals with a history of eating disorders and/or already low BMI/underweight and patients with specific medical conditions (e.g. diabetes treated with certain medications). The bone health of these subgroups requires special attention, and IF practices may, in theory, exacerbate/result in nutritional deficiencies and interact with growth/development or drugs with further implications for their bone health.
- *Dietary intervention characteristics*: The magnitude of the energy deficit elicited, the dietary composition of the intervention arms and the control of related lifestyle behaviours such as physical activity and sleeping patterns may all impact bone health parameters; nevertheless thus far, they are often poorly controlled and/or reported. For example, TRE regimens place emphasis on the duration and/or the timing of the eating window within a 24-h cycle and often disregard energetic intake or the quality of the diet consumed over the eating windows, which could introduce considerable variability in the bone responses. Similarly, the control arms are commonly instructed to follow their habitual diet which may significantly differ among individuals.

## Mechanistic perspectives

**Fig. 2.**
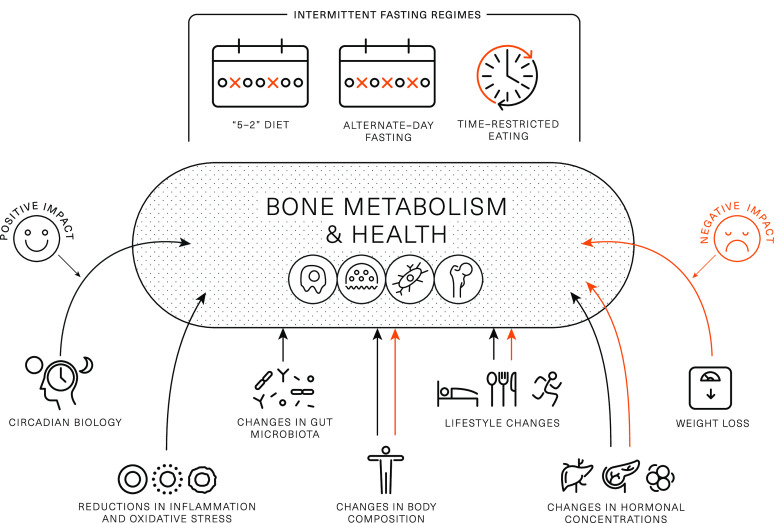
Theoretical framework on intermittent fasting characteristics and induced changes that may positively or negatively affect bone metabolism and health. Black arrows indicate positive impact, while red arrows indicate negative impact.

From a skeletal health perspective, IF interventions are very interesting as they involve behaviour/lifestyle changes and induce metabolic changes (e.g. realignment with circadian rhythm, changes in body weight, body composition, endocrine profile and gut microbiome) that theoretically may have positive, neutral or negative effects on bone health outcomes and their net effects are uncertain (Fig. 2).

## Components of energy balance and weight loss

IF typically leads to a reduction in energy intake^(5,6,21,34,40,42,68)^, which is likely the reason for weight loss, and current evidence suggests this is one of the primary mechanisms responsible for improvements in metabolic health^(3)^. Short-/medium-term continuous energy restriction (ranging in duration from a few days to 2–3 months) has been shown to promote bone breakdown (as assessed by BTMs)^(7,69–71)^, and longer-term (≥ 6 moths) continuous energy restriction is often accompanied by bone loss^(7,72)^. Thus, it is possible that IF causes a negative effect on bone health via a reduction in energy intake and accompanying weight loss. From the available literature, it is challenging to separate the effects of IF from those of weight loss. In Papageorgiou *et al.* ^(64)^, participants who lost weight by following standard dietary advice experienced small reductions in BMC, which were not seen in those who lost weight after a TRE intervention. In contrast, in another RCT in which ADF led to a significant reduction in energetic intake, and rapid weight loss, a reduction in lumbar spine BMD was reported^(57)^. Given these conflicting results, further studies appropriately designed to differentiate the effects of weight loss from other IF characteristics are needed.

In addition to reductions in energy intake and resulting weight loss, some IF interventions may reduce physical activity levels, and this could elicit an independent-detrimental effect on bone health. For example, skipping breakfast for 6 week resulted in a reduction in daily physical activity energy expenditure of approximately 450 kcal/d^(40)^, with similar findings reported with other methods of severe energy restriction^(22)^. Moreover, Templeman *et al.* observed a reduction in physical activity energy expenditure of approximately 100 kcal/d during 3 weeks of ADF, while no such reductions were observed when the same energy deficit was induced through daily energy restriction^(30)^. These studies indicate that IF may independently reduce physical activity to a greater extent than other methods of energy restriction, which may indirectly confer negative effects to bone health via a reduction in mechanical loading^(73)^. Conversely, interventions that have combined IF with exercise have been proved feasible and suggest that participants are able to perform moderate- to high-intensity endurance or resistance exercise during extended periods of fasting^(32,67,74)^. As such, individuals should be encouraged to engage in their exercise routines or new programmes for maximising metabolic and musculoskeletal benefits.

## Changes in body composition

Muscle, fat and bone are biomechanically and molecularly interacting tissues^(14,75)^. From a biomechanical perspective, it is well established that during locomotion and systematic exercise skeletal muscle applies forces on bone that stimulate high-magnitude strains which induce adaptations of bone mass, structure and strength. Furthermore, muscle and fat as contributors to body weight offer mechanical stimuli for increasing bone mass to support a higher body weight, while absolute reductions in muscle/fat and the resulting mechanical unloading have been proposed to partially explain the effects of weight loss on bone health^(13–15)^. The interactions between the three tissues at molecular level appear to involve (i) molecules produced by muscle (myokines such as IL6 and IL15, irisin) or fat (adipokines such as leptin and adiponectin) which act on bone, (ii) molecules secreted by bone (e.g. osteocalcin) with action on muscles/fat and iii) local/systemic endocrine factors (e.g. sex steroids) with effects on multiple tissues^(14,15,75)^.

Weight loss through energetic restriction derives largely from reductions in fat mass (accounting for ∼75 % of the weight lost) and to a less extent from fat-free mass loss (approximately the rest 25 %)^(76)^. The effects of IF regimens on body composition are still debated. Some reviews on this topic suggest reductions or no changes in fat mass and lean mass^(36)^ and a similar ratio of fat mass to lean mass loss (75–25 %) as conventional energy restrictions^(3)^. Conversely, some well-controlled studies have reported greater contributions of muscle mass loss to the total amount of weight loss^(30,61)^, raising the question whether IF interventions are safe for population groups at risk for osteoporosis (postmenopausal women, elderly and individuals with metabolic diseases) and skeletal injuries (e.g. athletes). To date, the contributions of body composition changes to changes in bone outcomes remain uncertain.

## Endocrine factors

Changes in body mass and composition cause changes in several tonic hormones implicated in bone metabolism and health. For example, insulin and leptin are known to have anabolic effects on bone; nevertheless, the influence of resistance/sensitivity to their actions on bone remains unclear. For example, obesity is associated with high insulin and leptin concentrations, which are thought to contribute to the higher BMD values seen in individuals with overweight/obesity^(15,77)^. Nevertheless, hyperglycaemia, excess insulin levels and insulin resistance (i.e. in type 2 diabetes) are purported to be associated with low bone turnover, impaired bone microstructure and bone matrix quality and thus, increased fracture risk^(19,77)^. Conversely, weight loss enhances insulin and leptin resistance but reduces their absolute concentrations; these changes appear to be associated with weight-induced bone loss^(13,15)^. Available research demonstrates that IF interventions result in reductions in fasting blood glucose and insulin, improvements in insulin sensitivity and decreases in leptin levels^(78)^; however, the impact of these changes on bone outcomes remains unexplored.

Insulin-like growth factor-1 (IGF-1) is another important anabolic factor for bone^(79)^, with current studies suggesting no changes or decreases in IGF-1 circulating levels in response to energetic and/or protein restrictions over periods 6–24 months^(10,80–82)^. IF studies have reported mixed results on IGF-1 responses. For example, an intervention of TRE (8-h eating window) in conjunction with resistance training resulted in decreases in testosterone and IGF-1 levels; nevertheless, these changes were not accompanied by unfavourable changes in body composition or compromises of muscle strength at least over the timeframe of the study. In contrast, in an ADF intervention, IGF-1 was unaltered in the ADF and the control groups but increased after continuous energy restriction, with no changes seen in bone mass in any of the three intervention groups^(60)^.

Many of the endocrine changes discussed are likely due to energy/macronutrient restriction and weight loss, rather than IF specifically. However, a distinguishing feature of IF is the frequent metabolic shift that occurs, owing to the switch between the prolonged catabolic fasted state and shortened anabolic periods of feeding. Prolonging the catabolic state stimulates lipid turnover more than traditional daily energy restriction, causing a proportional increase in lipid metabolism and a reciprocal reduction in the carbohydrate metabolism^(28)^. There have been several benefits to metabolic health proposed in relation to this switch, but a consequence is an acute period of post-prandial insulin resistance in response to the first meal consumed after breaking the fast^(28,40,83,84)^. Acutely elevated insulin concentrations have been shown to suppress concentrations of CTX and osteocalcin^(85)^.

Several gastrointestinal hormones (e.g. ghrelin, peptide YY, glucagon-like peptide 1 and peptide-P) have shown acute changes upon transitioning from a prolonged catabolic to an anabolic state^(21,84)^. Differences in bone remodelling have been found when providing nutrients orally or intravenously, suggesting a mediating role of gastrointestinal hormones in bone turnover^(86)^. Incretin hormones, such as lucose-dependent insulinotropic polypeptide (GIP) and glucagon-like peptide 1 (GLP-1), enhance insulin secretion^(87)^, so they may influence bone health through insulin-mediated pathways^(77)^.

## Changes in dietary factors

A balanced diet with adequate intakes of certain nutrients (i.e. calcium (Ca) and proteins) and foods and food groups (e.g. dairy products, fruits and vegetables) is important for maximising and maintaining bone properties^(88)^. Conversely, an unbalanced Western type diet typically high in processed foods, saturated fats, refined sugars and salt appears to compromise bone health through direct (e.g. salt-induced increases urinary Ca excretion) and indirect (chronic inflammation, contribution to obesity and associated metabolic diseases) mechanisms^(89)^. Notably, individuals who follow IF regimens commonly place their focus on meal timing rather than food quantity or quality. Thus, IF interventions do not necessarily translate into a (bone) healthy diet. Several studies have reported changes in aspects of dietary quality during IF; nevertheless, these have not been characterised in relation to bone health. Research on macronutrient composition with ADF/the ‘5–2 diet’/TRE regimens does not support pronounced differences in carbohydrate, protein or fat intake (as % of energy intake) pre- and post-interventions (for a review, see^(3)^), although reductions in absolute amounts of macronutrients appear to contribute to the lower energy intakes reported. Yet, a pertinent question, especially for individuals at risk for bone loss and muscle wasting, is whether IF protocols offer opportunities for meeting protein recommendations^(90)^. Current evidence supports additional musculoskeletal benefits from higher protein intakes (≥ 1·2 g/kg/d) for older individuals, while, to maximise protein synthesis, distribution of protein intake over waking hours and consumption of ≥ 2 meals per day with ∼0·4 g protein/kg are encouraged^(91,92)^. Such recommendations appear somewhat discordant with IF protocols in which all energy content are consumed within a shortened period of time each day (in the case of TRE) or are severely restricted for periods > 24 h (in case of ADF and the *5:2* diet). Whether IF regimens result in changes in micronutrient intakes and specific food group intake with subsequent implications for bone health remains unknown. Hypothetically, if somebody habitually consumes a breakfast rich in dairy products and this meal is skipped as part of practising TRE, this person may miss the opportunity to consume adequate Ca intake. Conversely, positive eating behaviour changes such as reductions in late evening snacks and alcohol observed after TRE^(37)^ may have favourable influences on bone health^(93,94)^.

## Circadian biology

Several lines of evidence suggest that bone is subjected to circadian variability (for a review, see^(20)^). Clock genes are expressed in bone cells, while clock gene knockout mice exhibit altered bone phenotypes. In line with this preclinical evidence, clinical studies have shown that bone-related hormones and BTM display circadian variations, while circadian rhythms disturbances such as working night shifts and/or sleeping disorders have been associated with impaired bone metabolism, reduced bone mass and increased fracture risk^(20)^. Conversely, it remains largely unknown how alignment of mealtimes with circadian rhythms such as those achieved in TRE may impact bone health. Indirect evidence suggests that favourable changes in circadian biology as a result of TRE are linked to cardiometabolic benefits which may occur independently of weight loss^(16,44)^ and which have been linked to improved bone outcomes in separate investigations^(18,95)^. The direct links between measurable TRE-induced circadian changes and bone health outcomes require elucidation.

## Changes in gut microbiome

Changes in gut microbiota during IF are important mediators of its metabolic benefits^(96)^. Preclinical studies have shown that fasting periods induce a ‘gut rest’ which contributes to (i) improved gut barrier function (e.g. increased villi length and expression of tight junction proteins^(97,98)^ and reductions in plasma levels of lipopolysaccharide^(99)^), (ii) enhanced gut microbial richness^(100)^, (iii) enrichment of beneficial bacteria^(97–99,101)^, (iv) alteration in microbial pathways involved in fuel utilisation (e.g. up-regulation of ketone body pathway), antioxidant signalling (enhancement of glutathione metabolism pathways) and low-grade inflammation (down-regulation of the lipopolysaccharide biosynthesis pathway)^(99)^ and (v) changes in gut microbiota-associated metabolites (e.g. increases in faecal short-chain fatty acids (SCFAs))^(97)^. There are limited studies in humans that have assessed such parameters, but of those few, some confirm some beneficial gut microbiota changes after TRE^(102)^ or IF during Ramadan^(103,104)^, while others have shown no significant alterations^(105)^. Research suggests that the gut microbiome affects bone health through several mechanisms including the production of metabolites (e.g. SCFAs) that affect bone metabolism, the bioavailability of nutrients important for bone health (e.g. Ca), the regulation of the immune system and hormonal modulation^(106,107)^. Given this emerging evidence of the gut–bone axis positive effects on gut microbiota may, in theory, positively affect the skeleton. Future animal and human studies need to address the complex interactions between different IF regimens, gut microbiota and bone health outcomes.

### Conclusions

IF represents a promising dietary approach for weight loss and prevention/treatment of metabolic disorders; nevertheless, its effects on bone health have only recently started to be unravelled. While animal studies currently offer limited insights into scenarios pertinent to humans and epidemiological studies are largely lacking from this area of research, most available evidence comes from interventional studies that have reported bone outcomes. These suggest that TRE regimens practised up to 6 months do not adversely affect bone outcomes and may have small protective bone effects when modest weight loss is achieved (< 5 % of baseline body weight). Similarly, most current research on ADF has shown no adverse effects on bone outcomes, while no studies on *the ‘5–2’ diet* have assessed bone outcomes. Available studies are limited by their short duration (3 weeks to 6 months), their small and diverse population samples, assessment of bone mass exclusively by total body DXA and inadequate control of factors that may affect bone outcomes. Thus, the interpretation of existing findings is challenging, and further research is required to better characterise bone responses to various IF schedules using well-controlled protocols of longer duration, adequately powered to assess changes in bone outcomes and designed to include clinically relevant bone assessments (BMD at the hip/lumbar spine, bone microstructure and fracture risk).
